# Compliance with COVID-19-preventive behaviours among employees returning to work in the post-epidemic period

**DOI:** 10.1186/s12889-022-12709-9

**Published:** 2022-02-21

**Authors:** Jie Liu, Yan Tong, Shaoqiong Li, Zhiqiang Tian, Lu He, Jianzhong Zheng

**Affiliations:** 1 Department of School Medicine,School of Public Health, Shanxi Medical University, Taiyuan, 030001 Shanxi China; 2 Department of Infectious Disease Control, Center for Disease Control and Prevent, Shizuishan, China; 3 Center for Information, Chinese Center for Disease Control and Prevention, Beijing, 102206 China

**Keywords:** Compliance with COVID-19-preventive behaviours, Coronavirus, COVID-19, Post-epidemic period, Preventive behaviours

## Abstract

**Background:**

The COVID-19 pandemic has underscored the importance of behaviours such as social distancing in controlling pandemics. Currently, the epidemic is under control in China and production has resumed in various industries. This study investigates the behavioural compliance and related factors for COVID-19 prevention among employees returning to the workplace and provide strategic recommendations for improving individual-level preventive behaviour to prevent a new outbreak.

**Methods:**

A cross-sectional study design was used. Data were gathered from returning employees in China using an online questionnaire survey, from March to May, 2020. The questionnaire covered participants’ COVID-19-related knowledge, compliance with recommended preventive behaviours, and levels of depression and anxiety. Univariate and multi-factor methods were used to analyse the data and identify factors influencing behaviour compliance.

**Results:**

Of the 1300 participants completing the full survey, more than half were male (71.92%) and 61% were aged between 31 and 50 years. Six hundred and ninety-eight (53.7%) participants showed high compliance, while 602 (46.3%) showed low compliance. In models adjusted for demographic and socio-economic factors, high education level (odds ratio [OR] = 0.23, 95% confidence interval [CI]: 0.07–0.70), office staff (OR = 0.51, 95% CI: 0.33–0.78), higher knowledge of COVID-19 (OR = 0.74, 95% CI: 0.67–0.81), and quarantining (OR = 0.74, 95% CI: 0.57–0.96) predicted better compliance with preventive behaviours (*P* <  0.05), while high anxiety levels (OR = 1.55, 95% CI: 1.10–2.18) predicted lower compliance with preventive behaviours (*P* <  0.05).

**Conclusion:**

For employees returning to work during the post-COVID-19-epidemic period, compliance with recommended preventive behaviours requires improvement. Consequently, comprehensive intervention measures, including the provision of health education and psychological counselling, as well as the continuance of a strict isolation policy, could enhance such compliance.

**Supplementary Information:**

The online version contains supplementary material available at 10.1186/s12889-022-12709-9.

## Background

Novel coronavirus (COVID-19) pneumonia is an emerging infectious disease caused by severe acute respiratory syndrome coronavirus 2, and is characterised by high infectivity and complex transmission routes [1, 2]. On 11 March 2020, the World Health Organization (WHO) defined COVID-19 as a ‘global pandemic’ [3]. There is growing evidence that effective person-to-person transmission of COVID-19 occurs in asymptomatic individuals, even during the incubation period [4, 5]. Departments at all levels in China promptly implemented measures on the prevention and control of the outbreak of COVID-19 pneumonia. They followed President Xi Jinping’s mandate: ‘When an epidemic breaks out, a command is issued. It is our responsibility to prevent and control it’ [6].

Many public health measures were also implemented; for example, during the pandemic, individuals with a history of travel to or residence in high-risk areas or countries (where continuous transmission of local cases has been identified) were asked to undergo 14 days of self-isolation in dedicated facilities [7]. All individuals were required to have their temperature monitored, and those exhibiting any flu-like symptoms were separated from their family members to carry out further quarantine measures. Great changes have also taken place in people’s behaviours and habits. People were instructed to wear masks in public places and to keep at least one metre distance from each other.

Consequently, as the COVID-19 epidemic was brought under control, the entire country began to resume production, and society began to return to normal in early 2020. However, the question of how to retain this level of progress remains. The WHO has recommended that people wear masks, maintain a minimum physical distance of at least one metre from others, avoid gathering in large crowds, and avoid eating meat from wild animals [8, 9]. Such behaviours can reduce the risk of infection, as the increased spread of COVID-19 is closely related to a limited adoption of preventive behaviours [10].

There is substantial evidence that transmission is closely related to a person’s socio-economic position [11, 12]; however, less attention has been paid to the factors associated with post-epidemic preventive behaviour. In the post-epidemic period, behavioural factors will be crucial to ensure that the epidemic does not return [13, 14]. Studies have shown that ‘behavioural fatigue,’ arising from quarantining or locking down too early, may lead to widespread non-adherence over time [15]. Therefore, this study aimed to (1) examine existing preventive behaviours, (2) test the relationship among behaviours that are embedded in complex systems involving individuals and socio-economic factors, and (3) provide strategic recommendations for improving individual-level preventive behaviour in the context of the prevention and control of the epidemic after returning to work in the post-epidemic period.

## Methods

### Participants and procedure

From 15 March to 15 May 2020, a questionnaire survey was administered to employees in China who were returning to work after the COVID-19 pandemic through an online platform (https://www.wjx.cn/app/survey.aspx). The participants were presented with a description of the study and were informed that participation was voluntary and anonymous. The inclusion criteria were: Older than 18 years old, returning to work between 15 March and 15 May 2020, and able to independently complete the online questionnaire. The exclusion criteria were: Younger than 18 years old (minor), not working or not yet returned to work, having a confirmed or suspected COVID-19 infection after resuming work or during isolation, and being unable to read or write, or to complete the questionnaire independently.

Invitations were sent to all potential participants via WeChat, the most popular social media app in Mainland China with one billion active users daily. At the same time, the research team providing medical services in the quarantine area and in the community offered to scan the QR code on the facility to complete the online survey.

Online informed consent was obtained by asking participants to check the box on their device’s screen with the response of their choice (i.e. ‘I agree to participate in the survey’ or ‘I do not agree to participate in the survey’). If they checked ‘I do not agree’, the computer program terminated automatically. The questionnaire and survey was specifically developed for this study. The full questionnaires are presented in Additional file 1.

The study was approved by the institutional review board of Ningxia Medical University (document number: 2020112).

### Survey instrument

#### Socio-demographic information

Part of our online questionnaire consisted of questions concerned with socio-demographic information, including sex, age, marital status, education level, household registration, occupation, years of employment, quarantine status, and source of epidemic-related concerns.

#### COVID-19 knowledge

Based on the New Diagnosis and Treatment Scheme for Novel Coronavirus Infected Pneumonia (fifth trial edition) [16] and the Protocol on Prevention and Control of COVID-19 (sixth edition) [17] issued by the National Health Commission of China, we developed a definitive questionnaire concerning the epidemiological characteristics, clinical symptoms, and comprehensive prevention and control measures for COVID-19. The scale comprised 10 items, with one point awarded for each correct answer (total score: 0–10). The higher the score, the higher the COVID-19 knowledge level.

#### Mental-health status

Considering the high incidence of depression and anxiety symptoms reported among quarantined subjects in previous studies [18], depression and anxiety were assessed in the present study, using the Patient Health Questionnaire (PHQ-9) and the Generalized Anxiety Disorder (GAD-7). Both scales have shown good reliability and validity in both foreign and domestic studies [19, 20]. Both scales use a four-point Likert scale (range: 0–3) for each item. For the PHQ-9, which comprises nine items, scores of 0–4, 5–9, 10–14, 15–19, and 20–27 indicate no, mild, moderate, severe, and extremely severe depression, respectively. The GAD-7 comprises seven items, with scores of 0–4, 5–9, 10–14, 15–21 indicating no, mild, moderate, and severe anxiety, respectively.

#### Behavioural compliance

To evaluate the respondents’ compliance with COVID-19-preventive behaviours when returning to work, items concerning 10 such behaviours were included. The 10 behaviours were 1) wearing masks; 2) covering your mouth and nose when coughing and sneezing; 3) washing your hands frequently; 4) avoiding contact with, buying, or eating the meat of wild animals; 5) being mindful of symptoms such as fever and coughing and performing comprehensive health monitoring; 6) avoiding close contact with people showing symptoms of respiratory disease; 7) avoiding crowded places; 8) keeping rooms clean and opening windows for ventilation; 9) reducing social gatherings and visits to friends and relatives; and 10) maintaining a healthy diet and taking moderate exercise. For these items, a five-point Likert scale was used (1 = ‘completely unnecessary’, 5 = ‘very necessary’). Total scores ranged from 5 to 50 points. Scores of ≥40 indicated higher compliance; scores of < 40 indicated lower compliance; this means that the lower the score is, the lower the behaviour compliance is.

### Quality control

As the questionnaire was electronic, responses were required for all questions before submission; that is, if the questions were not answered completely, they could not be submitted. A ‘skip item’ option was provided to increase the likelihood of obtaining complete and logical responses. To avoid repeat answers, each IP address could only access the questionnaire once. The survey took approximately 8–15 min to complete; a preliminary survey showed that it would take at least 300 s to complete all questions. If the questionnaire is completed in less than 300 s, it indicates that the participants did not read the questionnaire carefully, and that the quality of the responses will be poor; thus, the responses provided in less than 300 s were excluded.

### Data analysis

The data were checked and categorised. SPSS version 21.0 statistical software was used for statistical calculations. Two-sided *p*-values of 0.05 were considered to indicate statistical significance. Categorical variables are presented as frequencies and percentages, while continuous variables are presented as means and standard deviations (±SD). The percentage differences in behavioural compliance across categorical variables were examined using chi-squared tests. An unconditional logistic regression method was adopted to determine the factors influencing respondents’ compliance after controlling for covariates. We used the unconditional logistic analysis method, as the dependent variable is dichotomous and matching design data is not used in this study. The dependent variable was the level (higher/lower) of respondents’ compliance with preventive behaviours (1 = no, 0 = yes). Education level, occupation, COVID-19 knowledge, anxiety, and quarantining were set as independent variables, while gender, age, household registration, and years of employment were set as covariates. Multiple categorical variables (age, occupation, education, and years of employment) were set as dummy variables.

The adjusted odds ratios (OR) for the variables and their 95% confidence intervals (95% CIs) were calculated using the unconditional logistic regression model.

## Results

### Participants’ characteristics

As shown in Table 1, more than half of the participants were male (71.92%), were aged between 31 and 50 years (61%), and were living in rural areas (58.4%). Most participants were married (77.8%). Approximately one third of the participants (41.3%) had an educational qualification of junior college or above. Nearly half the participants had been quarantined (49.3%).

**Table 1:** Socio-demographic characteristics, scores of knowledge, anxiety, and depression associated with COVID-19, and behaviour compliance of participants

Variable	***N***(%)	COVID-19 knowledge M (±SD)	Anxiety M (±SD)	Depression M (±SD)	Behaviour M (±SD)
**Age (years)**					
18–25	42 (3.2)	7.88 (±1.37)	1.5 (±2.68)	1.93 (±3.11)	35.81 (±6.45)
26–30	187 (14.4)	8.04 (±1.27)	1.5 (±2.52)	2.56 (±3.83)	37.05 (±5.89)
31–40	355 (27.3)	8.14 (±1.22)	1.68 (±2.68)	2.43 (±3.54)	36.49 (±6.3)
41–50	438 (33.7)	8.05 (±1.3)	1.38 (±2.38)	1.95 (±3.14)	35.95 (±7.34)
51–60	226 (17.4)	7.97 (±1.35)	1.33 (±2.39)	2.05 (±3.39)	36.31 (±7.07)
Over 60	52 (4.0)	8.46 (±1.24)	1.54 (±3.06)	1.37 (±2.92)	37.83 (±10.45)
**Gender**					
Male	935 (71.9)	8.02 (±1.31)	1.49 (±2.59)	2.20 (±.348)	35.96 (±6.86)
Female	365 (28.1)	8.21 (±1.20)	1.46 (±2.35)	2.07 (±3.19)	37.49 (±7.09)
**Marital status**					
Unmarried	215 (16.5)	8.13 (±1.30)	1.27 (±2.33)	2.23 (±3.33)	38.20 (±6.85)
Married	1011 (77.8)	8.09 (±1.27)	1.52 (±2.57)	2.14 (±3.42)	36.09 (±6.91)
Divorced or widowed	74 (5.7)	7.73 (±1.38)	1.53 (±2.42)	2.24 (±3.38)	35.18 (±7.12)
**Education level**					
Junior middle school or below	369 (28.4)	7.62 (±1.35)	1.31 (±2.25)	1.81 (±3.08)	34.54 (±7.16)
High school or technical secondary school	394 (30.3)	8.00 (±1.29)	1.52 (±2.61)	2.22 (±3.51)	35.89 (±6.66)
Junior college	512 (39.4)	8.42 (±1.14)	1.58 (±2.66)	2.37 (±3.54)	37.93 (±6.66)
Bachelor’s degree or above	25 (1.9)	8.68 (±0.85)	1.24 (±2.22)	2.16 (±2.75)	39.92 (±6.77)
**Household registration**					
Rural area	759 (58.4)	8.16 (±1.26)	1.51 (±2.62)	2.28 (±3.51)	36.36 (±6.84)
Urban area	541 (41.6)	7.94 (±1.31)	1.44 (±2.39)	1.99 (±3.23)	36.43 (±7.13)
**Occupation**					
Outdoor worker	681 (52.4)	7.94 (±1.32)	1.45 (±2.53)	2.09 (±3.44)	35.39 (±6.57)
Office staff	154 (11.9)	8.33 (±1.24)	1.4 (±2.33)	2.31 (±3.42)	38.23 (±7.34)
Managerial or technical personnel	249 (19.1)	8.35 (±1.12)	1.71 (±2.58)	2.38 (±3.51)	38.1 (±7.7)
Other	216 (16.6)	8.00 (±1.33)	1.37 (±2.58)	2.05 (±3.12)	36.23 (±6.36)
**Years of employment**					
< 1	142 (10.9)	7.83 (±1.29)	1.27 (±2.33)	2.40 (±3.43)	35.8 (±6.59)
1–5	517 (39.8)	8.03 (±1.31)	1.44 (±2.48)	2.16 (±3.52)	36.4 (±6.34)
6–10	240 (18.5)	8.18 (±1.11)	1.53 (±2.36)	2.07 (±2.94)	37.88 (±7.45)
> 10	401 (30.9)	8.14 (±1.34)	1.57 (±2.74)	2.13 (±3.49)	35.68 (±7.41)
**Quarantine**					
No	659 (50.7)	8.07 (±1.27)	1.64 (±2.75)	2.35 (±3.66)	35.38 (±5.91)
Yes	641 (49.3)	8.07 (±1.30)	1.32 (±2.26)	1.97 (±3.09)	37.42 (±7.76)
**Source of epidemic-related concerns**					
Government release	726 (55.8)	8.10 (±1.28)	1.32 (±2.46)	2.00 (±3.29)	36.07 (±7.20)
The media	574 (44.2)	8.04 (±1.30)	1.68 (±2.60)	2.37 (±3.52)	36.79 (±6.62)
**Anxiety**					
No	1118 (86.0)	8.08 (±1.27)	0.60 (±1.10)	0.82 (±1.27)	36.52 (±6.92)
Yes	182 (14.0)	8.01 (±1.39)	6.86 (±2.09)	8.06 (±3.53)	35.60 (±14.0)
**Depression**					
No	1059 (81.5)	8.07 (±1.27)	0.68 (±1.43)	1.27 (±2.11)	36.47 (±6.95)
Yes	241 (18.5)	8.06 (±1.34)	4.98 (±3.24)	7.63 (±4.53)	36.03 (±6.96)
**Behaviour**					
< 40	602 (46.3)	7.75 (±1.36)	1.63 (±2.77)	2.35 (±3.66)	30.86 (±5.38)
≥ 40	698 (53.7)	8.35 (±1.15)	1.35 + 2.28)	2.00 (±3.15)	41.16 (±4.01)
**Total**	1300 (100.0)	8.07 (±1.29)	1.48 (±2.53)	2.16 (±3.40)	36.39 (±6.96)

### Respondents’ knowledge and psychological status

The respondents showed high awareness of basic COVID-19-related knowledge and most were aware of the associated protective actions; however, some areas required strengthening. The average score for the general knowledge questionnaire was 8.07 (±1.29); 971 (74.4%) participants had a total knowledge score ≥ 8 and 329 (25.3%) had a total knowledge score < 8. The three items with the lowest rate of correct responses were related to clear sources of infection (553 correct answers; 42.5%), clear routes of transmission (963; 74.1%), and clear symptoms of infection (651; 50.1%). The average score for the PHQ-9 was 2.16 (±3.40); 1059 (81.5%) participants showed no depression symptoms, while 241 (18.5%) showed signs of depression; 93 (14.8%), 35 (2.7%), 9 (0.7%), and 4 (0.3%) had mild, moderate, severe, and very severe depression, respectively. The average GAD-7 score was 1.48 (±2.53), with 1118 (86.0%) showing no anxiety and 182 (14.0%) showing anxiety; 167 (12.8%), 13 (1%), and two (0.2%) had mild, moderate, and severe anxiety, respectively. A Spearman’s rank correlation analysis showed a strong correlation between anxiety and depression, *R* = 0.693 (*P* <  0.001).

### Compliance with preventive behaviours

The average score for compliance with preventive behaviours was 36.39 (±6.96); 698 (53.7%) participants showed higher compliance (total score ≥ 40) and 602 (46.3%) showed lower compliance (total score < 40). The three behaviours with the worst compliance were covering your mouth and nose when coughing or sneezing (50.84%), keeping rooms clean and opening windows for ventilation (54.11%), and avoiding crowded public places (56.92%).

### Behavioural compliance in terms of demographic and socio-economic factors characteristics

In terms of behavioural compliance, as shown in Table 2, there were no statistically significant differences regarding age, household registration, source of epidemic-related concerns, or presence of signs of depression. However, statistically significant differences were found for gender, marital status, education level, occupation, years of employment, quarantining, and presence of anxiety (*P* <  0.05; Table 2). A Spearman’s rank correlation analysis was performed on both knowledge and behaviour scores; a value of *P* <  0.001 was obtained, indicating that knowledge and behaviour were significantly correlated.

**Table 2:** Comparison and analysis of participants’ compliance with preventive behaviours and their demographic characteristics

Variable	Grouping	Higher compliance ***N*** (%)	Lower compliance ***N*** (%)	χ^**2**^	***P*** value
Age (years)	18–25	23 (1.8)	19(1.4)	6.535	0.258
	26–30	107 (8.2)	80(6.2)		
	31–40	206 (15.9)	149 (11.5)		
	41–50	220 (16.9)	218 (16.8)		
	51–60	115 (8.9)	111 (8.5)		
	Over 60	27 (2.1)	25 (1.9)		
Gender	Male	481 (37.0)	454 (34.9)	6.771	0.009
	Female	217 (16.7)	148 (11.4)		
Marital status	Unmarried	142 (10.9)	73 (5.6)	16.71	< 0.001
	Married	522 (40.2)	489 (37.6)		
	Divorced or widowed	34 (2.6)	40 (3.1)		
Education level	Junior middle school or below	141 (10.9)	228 (17.5)	81.916	< 0.001
	High school or technical secondary school	195 (15.0)	199 (15.3)		
	Junior college	341 (26.2)	171 (13.2)		
	Bachelor’s degree or above	21 (1.6)	4 (0.3)		
Household registration	Rural area	418 (32.2)	341 (26.2)	1.391	0.237
	Urban area	280 (21.5)	261 (20.1)		
Occupation	Outdoor worker	318 (24.5)	363 (27.9)	55.537	< 0.001
	Office staff	110 (8.5)	44 (3.4)		
	Managerial or technical personnel	168 (12.9)	81 (6.2)		
	Other	102 (7.8)	114 (8.8)		
Years of employment	< 1	67 (5.1)	75 (5.8)	13.757	0.003
	1–5	279 (21.5)	238 (18.3)		
	6–10	152 (11.7)	88 (6.8)		
	> 10	200 (15.4)	201 (15.5)		
Quarantine	No	320 (24.6)	339 (26.1)	14.168	< 0.001
	Yes	378 (29.1)	263 (20.2)		
Source of epidemic-related concerns	Government release	376 (28.9)	350 (26.9)	2.392	0.122
	The media	322 (24.8)	252 (19.4)		
Anxiety	No	615 (47.3)	503 (38.7)	5.568	< 0.001
	Yes	83 (6.4)	99 (7.6)		
Depression	No	578 (44.5)	481 (37.0)	1.81	0.179
	Yes	120 (9.2)	121 (9.3)		

### Analysis of factors influencing compliance with preventive behaviours

As revealed in Table 3, education level, occupation, experience of quarantine, COVID-19 knowledge, and anxiety influenced participants’ compliance in adopting preventive behaviours. After controlling for covariates (age, educational level, household registration, and years of employment), those with bachelor’s degrees or above (OR = 0.23, 95% CI: 0.07–0.70); office staff (OR = 0.51, 95% CI: 0.33–0.78); people who were quarantining (OR = 0.74, 95% CI: 0.57–0.96); and those with good knowledge of COVID-19 (OR = 0.74, 95% CI: 0.67–0.81) all showed better behavioural compliance than their counterparts. Finally, people with anxiety showed lower behavioural compliance (OR = 1.55, 95% CI: 1.10–2.18).

**Table 3:** Univariate and multivariate analyses of compliance

Variables	OR (95% CI)	*P* value	aOR^a^ (95% CI)^a^	*P* value
Age (years)				
18–25	Ref		Ref	
26–30	0.91 (0.46–1.77)	0.771	1.26 (0.62–2.60)	0.516
31–40	0.88 (0.46–1.67)	0.686	1.12 (0.56–2.32)	0.686
41–50	1.20 (0.64–2.27)	0.575	1.40 (0.69–2.84)	0.348
51–60	1.17 (0.60–2.26)	0.645	1.32 (0.62–2.80)	0.468
Over 60	1.12 (0.50–2.53)	0.784	1.94 (0.76–4.93)	0.165
Gender				
Male	Ref		Ref	
Female	0.72 (0.57–0.92)	0.09	0.94 (0.71–1.23)	0.634
Household registration				
Rural area	Ref		Ref	
Urban area	1.14 (0.92–1.43)	0.237	0.98 (0.74–1.28)	0.862
Education level				
Junior middle school or below	Ref		Ref	
High school or technical secondary school	0.63 (0.47–0.84)	0.002	0.71 (0.52–0.98)	0.036
Junior college	0.31 (0.24–0.41)	< 0.001	0.49 (0.35–0.70)	< 0.001
Bachelor’s degree or above	0.12 (0.04–0.35)	<0.001	0.23 (0.07–0.70)	0.010
Occupation				
Outdoor worker	Ref		Ref	
Office staff	0.35 (0.24–0.51)	< 0.001	0.51 (0.33–0.78)	0.002
Managerial or technical personnel	0.42 (0.31–0.57)	< 0.001	0.69 (0.49–0.98)	0.037
Other	0.98 (0.72–1.33)	0.893	1.16 (0.83–1.61)	0.386
Years of employment				
< 1	Ref		Ref	
1–5	0.76 (0.53–1.11)	0.152	0.90 (0.61–1.36)	0.634
6–10	0.52 (0.34–0.79)	0.002	0.71 (0.45–1.14)	0.156
> 10	0.90 (0.61–1.32)	0.581	1.09 (0.69–1.70)	0.722
Quarantine				
No	Ref		Ref	
Yes	0.66 (0.53–0.82)	< 0.001	0.74 (0.57–0.96)	0.023
Anxiety				
No	Ref		Ref	
Yes	1.46 (1.07–2.00)	0.019	1.55 (1.10–2.18)	0.012
COVID-19 knowledge	0.69 (0.63–0.75)	< 0.001	0.74 (0.67–0.81)	< 0.001

*Ref* Reference category, *OR* odds ratio, *aOR* adjusted odds ratio, *CI* confidence interval

^a^Adjusted for gender, age, educational level, Household registration and years of employmentlis

## Discussion

Since the outbreak of COVID-19, China has implemented comprehensive government and social mobilisation, and the epidemic has gradually been brought under control [21]. This study involved a comprehensive investigation of the COVID-19-related knowledge, attitudes, and behaviours of personnel returning to work in the post-epidemic period, focusing on identifying factors that influence compliance with preventive behaviours. The behaviour compliance of nearly half of the respondents is not ideal, mainly in terms of ‘covering your mouth and nose when sneezing’, ‘avoiding close contact with people with respiratory diseases’, and other behavioural problems. Education, occupation, quarantining, anxiety, and knowledge factors impacted respondents’ health behaviours. Based on the identification of the factors influencing compliance, we can propose targeted measures to boost preventive behaviours. Our findings can inform future policies to prevent the further spread of COVID-19 in China and globally, to mitigate the impact of adverse preventive behaviours. They can also provide a basis for epidemic prevention and control during the resumption of work and production and lay the foundation for smooth production, and life in general in all sections of the society, to prevent the recurrence of the epidemic.

In terms of socio-economic indicators, this study included two indicators: education and occupation. People with higher education are more likely to be employed, especially at higher levels or in more prominent positions in companies and organisations [22]. The present findings showed that the higher academic background, the higher knowledge of COVID-19, the better their compliance with preventive behaviours; this is consistent with previous research [23]. This may be because people with higher levels of education may have a greater sense of health self-awareness [24]. Recent studies reported that a higher education level predicts higher knowledge of COVID-19 [25], which is consistent with our study. This indicates that more comprehensive knowledge of COVID-19, especially regarding prevention and control measures and prognoses, leads to better behavioural compliance and a more active response to COVID-19 prevention and control measures. Concurrently, this study found that the respondents had a poor understanding of the sources of COVID-19 infection, the transmission routes, and the symptoms of infected people. Public information campaigns and educational interventions could improve the overall awareness of infectious-disease-prevention knowledge [26]. Thus, relevant government departments should strengthen knowledge dissemination regarding the epidemiological characteristics of COVID-19 and training regarding symptom recognition, especially for people with low education levels. Further, medical personnel, while conducting health-monitoring work, could contribute to this by issuing informational manuals or WeChat short videos.

The respondents’ occupations were also related to their compliance with preventive behaviours, with office and management personnel and professional and technical personnel reporting better scores than outdoor workers, which is in line with existing research [27, 28]. This may be because office staff have a more regular workplace and contact with the same people; hence, their behaviour is more compliant. Outdoor or out-of-office workers, however, are exposed to a greater risk of infection due to more contact with strangers who may be infected [29]. For example, face-to-face contact reduces social distance in tourism, transportation, and retail sectors [30]. Furthermore, most outdoor workers have low socio-economic status, and their education level is generally low, which often leads to low compliance [27, 31]. Therefore, government health departments and enterprises should focus on monitoring temperature, conducting computed tomography (CT) imaging and the nucleic acid test, and providing health education for outdoor workers with low socio-economic status. Businesses should conduct regular, scientific, and effective disinfection of workplaces, including the cleaning and disinfection of public areas such as offices, production workshops, vehicles, restaurants, elevators, meeting rooms, toilets, and object surfaces [32].

Existing studies have also shown that anxiety is common during epidemics, and that psychological conditions influence behavioural compliance [33]. In this study, people with high anxiety levels were found to be more likely to have poor compliance with preventive behaviours [34]. From this finding, it is clear that anxiety is linked to a lack of awareness and reluctance to perform recommended behaviours. This may arise from accepting misleading information on social media, leading to inadequate awareness of COVID-19–related prevention and control measures [22]. Businesses should be aware of employees’ mental-health status before they return to work, and should provide psychological consultation services, if necessary [22]. Further, false information on the Internet regarding COVID-19 can cause panic [35, 36]; therefore, in the short term, it is necessary to strengthen the management of online information and support scientific, in-depth, and continuous reporting by official media channels, which would serve to guide the direction of public opinion [37]; in the long term, public health literacy should be greatly improved [38].

The rationale for studying the relationship between quarantine and behavioural compliance is that a large proportion of individuals who are returning to work in China have experienced isolation or self-isolation; studies have also shown that lockdown or quarantine may induce ‘behavioural fatigue’ [39], thereby necessitating an examination of the compliance of people after quarantine. Our results showed that quarantined people had better compliance with preventive behaviours than did non-quarantined people. This indicates that people who have taken quarantine measures pay more attention to prevention and control behaviours, which plays a positive role in epidemic prevention and control. Research has shown that quarantining facilitates symptom surveillance, which allows early diagnosis and reduces the risk of infecting others [40, 41]. This approach also strategically supports subsequent epidemic prevention measures; therefore, the quarantine policy should continue to be strengthened. For those who have not been quarantined, publicity and education should be strengthened to improve compliance behaviour. According to our analysis, outdoor workers had the largest number of people in isolation (258, 40.25%, *P* < 0.001), thereby adding to the literature by providing evidence that outdoor personnel are exposed to more risk factors and thus should be the focus of outbreak prevention and control. Based on this finding, we recommend that during the isolation process, health education should be conducted through video courses, media, direct education. Information manuals for such prevention and control measures and a practical mental health manual could be developed [42], which could help employees improve their awareness of how to protect themselves. Finally, quarantine requires the collaboration of multiple organisations and agencies. Isolation not only requires the planning and implementation of the health sector and the cooperation of high-risk groups, it also requires the government and society to provide reasonable job security and social security for the quarantined group.

## Conclusions

In this study, a rapid population survey was conducted during the COVID-19 epidemic using online survey questionnaire. The knowledge, attitudes, and behaviours of people returning to work in the later period of the epidemic were determined, as were the factors influencing their behaviours. The findings can represent a reference for improving behavioural interventions, as well as forming a theoretical basis for epidemic prevention and control. The limitations of this study arise from the limitations of the online survey tool, wherein the independent variables were analysed focusing on objective general demographic characteristics, with fewer indicators reflecting participants’ socio-economic status; this could restrict the extrapolation of results.

## Supplementary Information

**Additional file 1: Supplementary materials.** A survey of COVID-19 prevention knowledge Behavior and Attitude among Employees returning to work.

## Data Availability

The data supporting the findings of this study are available from Ningxia Medical University. However, restrictions apply to the availability of these data, which were used under license for the current study, and so are not publicly available. Data are however available from the corresponding author upon reasonable request and with permission of Ningxia Medical University.

## Abbreviation

WHO: World Health Organization
