# Supplementary material for: Compliance with COVID-19-preventive behaviours among employees returning to work in the post-epidemic period
Source: BMC Public Health. 2022 Feb 21;22:369. doi: 10.1186/s12889-022-12709-9 (PMC8860284; doi:10.1186/s12889-022-12709-9)
Supplement: Supplementary file 1 — Additional file 1: Supplementary materials. A survey of COVID-19 prevention knowledge Behavior and Attitude among Employees returning to work. [file 12889_2022_12709_MOESM1_ESM.docx]

**Supplementary materials:**

**A survey of COVID-19 prevention knowledge Behavior and Attitude among Employees returning to work**

Hello!The COVID-19 outbreak in January 2020 has touched the hearts of hundreds of millions of people in the world, and the general public has been highly cooperative in epidemic prevention and control.This is an important period for enterprises to resume work and production, and also a critical period for the prevention and control of the epidemic.The purpose of this survey is to understand your health status and needs, and to provide scientific basis for further promoting the parallel and orderly progress of epidemic prevention and control and the resumption of work and production of enterprises, so as to provide effective support and help for you！
      This survey is conducted anonymously. We guarantee that the information you fill in will be kept confidential. All information will only be used for academic research purposes.This survey will take you about 10-15 minutes. Please read the following items carefully and complete the scale independently. Thank you for your support and cooperation!

**The basic information**

1.Your gender： [Single topic selection] *

| ○Male | ○Female |  |  |  |  |  |  |
| --- | --- | --- | --- | --- | --- | --- | --- |

2.Your age： [Single topic selection] *

| ○18~25 | ○26~30 | ○31~40 | ○41~50 | ○51~60 | ○Above60 |  |
| --- | --- | --- | --- | --- | --- | --- |

3.Your marital status [Single topic selection] *

| ○Unmarried | ○married | ○Divorced | ○Remarried | ○Death of a spouse |
| --- | --- | --- | --- | --- |

4.Your highest education [Single topic selection] *

| ○Without formal education | ○Primary school | ○Junior school | ○High school |
| --- | --- | --- | --- |
| ○Technical secondary school | ○Junior college | ○Bachelor degree | ○Graduate student |

5.Your household registration[Single topic selection] *

| ○Urban | ○Rural |
| --- | --- |

6.Your career： [Single topic selection] *

| ○Construction workers |
| --- |
| ○Production personnel |
| ○The sales staff |
| ○Marketing/pr staff |
| ○The personnel of the service |
| ○Administrative/Support personnel |
| ○The human resources |
| ○Financial/Auditor |
| ○Civilian/Clerical staff |
| ○Technical/Research and developmen personnel |
| ○Management personnel |
| ○Teachers |
| ○Consultant |
| ○Professionals (e.g. accountants, lawyers, architects, medical staff, journalists, etc.) |
| ○other |

7.Years of experience in this profession: [Single topic selection] *

| ○<1 Year | ○1-5 Years | ○6-10Years | ○>10Years |
| --- | --- | --- | --- |

8.For the past month, you have been placed under quarantine or medical observation at home [Single topic selection] *

| ○No | ○Yes |  |
| --- | --- | --- |

9.You follow the main source of COVID-19 epidemic information on a daily basis [Single topic selection] *

| ○The central government releases information | ○Local governments release information |
| --- | --- |
| ○Business news media (such as Sina and Tencent News) | ○We Media (such as Douyin, Kuaishou, etc.) |
| ○Acquaintances exchange information in private |  |

K1-10 **Questionnaire of knowledge towards COVID-19**

|  | Questions | Options |
| --- | --- | --- |
| 1 | Up to present, the main infection source was the patients who had been infected by the COVID-19. | True, false, I don’t know |
| 2 | Not all persons will be infected with COVID-19. Only those who are elderly, have chronic illnesses, and are obese are more likely to be infected. | True, false, I don’t know |
| 3 | People who have close contact with someone infected with the COVID-19 should be immediately quarantine in a proper place. In general, the observation period is 14 days. | True, false, I don’t know |
| 4 | The main route of transmission of COVID-19 is respiratory droplet transmission and it can also be transmitted through contact. | True, false, I don’t know |
| 5 | If you have suspicious symptoms regarding the COVID-19, you taking public transportation to the hospital without a mask and don’t stay away from others. | True, false, I don’t know |
| 6 | The main clinical symptoms of COVID-19 are fever, fatigue, dry cough, dyspnea etc., with or without nasal congestion, runny nose or other upper respiratory symptoms. | True, false, I don’t know |
| 7 | Visitors entering the affected areas,should take routine precautions including avoiding close contacts with people with acute respiratory infection, washing hands frequently and following appropriate coughing etiquette. | True, false, I don’t know |
| 8 | Community population can wear general medical masks to prevent the infection by the COVID-19 virus. | True, false, I don’t know |
| 9 | If you infected with the COVID-19, you can not go to the hospital and can heal yourself. | True, false, I don’t know |
| 10 | There is no specific drug treatment against the COVID-19 currently, but early symptomatic and supportive treatment can help most patients recover from the infection. | True, false, I don’t know |

XA1-10 The following is the behavior phase you are in, please select the option that fits your situation：*

| Do you think these quarantine measures below are necessary? | Very unnecessary | Unnecessary | Undecide | Necessary | Very necessary |
| --- | --- | --- | --- | --- | --- |
| 1. Choose your mask correctly and wear it. | □_1_ | □_2_ | □_3_ | □_4_ | □_5_ |
| 2. Cover your mouth and nose when you cough or sneeze. | □_1_ | □_2_ | □_3_ | □_4_ | □_5_ |
| 3. Wash your hands and keep them clean. | □_1_ | □_2_ | □_3_ | □_4_ | □_5_ |
| 4. Do not touch, buy or eat wild animals. | □_1_ | □_2_ | □_3_ | □_4_ | □_5_ |
| 5. Pay close attention to fever, cough and other symptoms, and do good health monitoring. | □_1_ | □_2_ | □_3_ | □_4_ | □_5_ |
| 6.Avoid close contact with people with respiratory symptoms such as fever, coughing or sneezing. | □_1_ | □_2_ | □_3_ | □_4_ | □_5_ |
| 7.Avoid crowded public places. | □_1_ | □_2_ | □_3_ | □_4_ | □_5_ |
| 8.Keep the room clean and open Windows frequently for ventilation. | □_1_ | □_2_ | □_3_ | □_4_ | □_5_ |
| 9.To reduce on visits and dinners. | □_1_ | □_2_ | □_3_ | □_4_ | □_5_ |
| 10.Pay attention to nutrition and exercise moderately. | □_1_ | □_2_ | □_3_ | □_4_ | □_5_ |

PHQ-9 Depressive Scale.*

| To what extent do you have the following questions in the last two weeks?(Please tick the appropriate box) | Not at all | Several days | More than half the days | Nearly every day |
| --- | --- | --- | --- | --- |
| 1. Lack of enthusiasm or interest in doing something | □_1_ | □_2_ | □_3_ | □_4_ |
| 2. To feel depressed, depressed, or hopeless | □_1_ | □_2_ | □_3_ | □_4_ |
| 3. Difficulty in falling asleep, staying asleep, or waking up | □_1_ | □_2_ | □_3_ | □_4_ |
| 4. Feeling tired or inactive | □_1_ | □_2_ | □_3_ | □_4_ |
| 5. I have no appetite and don't feel like eating | □_1_ | □_2_ | □_3_ | □_4_ |
| 6. Feeling terrible, a failure, or a disappointment to your family | □_1_ | □_2_ | □_3_ | □_4_ |
| 7.Difficulty concentrating, such as inability to concentrate on reading a newspaper or watching TV | □_1_ | □_2_ | □_3_ | □_4_ |
| 8.Speak or act very slowly, or, conversely, move around and fidget | □_1_ | □_2_ | □_3_ | □_4_ |
| 9.There are thoughts of dying or hurting yourself in some way | □_1_ | □_2_ | □_3_ | □_4_ |

GAD-7 Anxiety Scale. *

| Over the last 2 weeks, how often have you been bothered by the following problems? (Please tick the appropriate box) | Not at all | Several days | More than half the days | Nearly every day |
| --- | --- | --- | --- | --- |
| 1. Feeling nervous, anxious or on edge | □_1_ | □_2_ | □_3_ | □_4_ |
| 2. Not being able to stop or control worrying | □_1_ | □_2_ | □_3_ | □_4_ |
| 3. Worrying too much about different things | □_1_ | □_2_ | □_3_ | □_4_ |
| 4. Trouble relaxing | □_1_ | □_2_ | □_3_ | □_4_ |
| 5. Being so restless that it is hard to sit still | □_1_ | □_2_ | □_3_ | □_4_ |
| 6. Becoming easily annoyed or irritable | □_1_ | □_2_ | □_3_ | □_4_ |
| 7. Feeling afraid as if something awful might happen | □_1_ | □_2_ | □_3_ | □_4_ |
